# Associations between Vitamin D and Liver Function and Liver Fibrosis in Patients with Biliary Atresia

**DOI:** 10.1155/2019/4621372

**Published:** 2019-11-03

**Authors:** Peijun Zhuang, Song Sun, Rui Dong, Gong Chen, Yanlei Huang, Shan Zheng

**Affiliations:** Department of Pediatric Hepatobiliary Surgery, Children's Hospital of Fudan University and Key Laboratory of Neonatal Disease, Ministry of Health, 399 Wan Yuan Road, Shanghai 201102, China

## Abstract

**Objectives:**

To detail the effects of vitamin D (VD) deficiency and assess the relationships between VD deficiency and liver function and liver fibrosis in patients with biliary atresia (BA).

**Methods:**

In this study, BA patients confirmed by intraoperative cholangiography were enrolled between January 2017 and February 2019. Preoperative serum 25-(OH)D level, liver function, serum biomarker levels of liver fibrosis, and histopathologic features were recorded. Deficiency, insufficiency, and sufficiency of VD were defined as serum 25-(OH)D concentrations of <10, 10-20, and >20 ng/ml, respectively. Associations between serum 25-(OH)D level and liver function and liver fibrosis were analyzed.

**Results:**

A total of 161 BA infants were included. The median (interquartile range (IQR)) serum 25-(OH)D level in all patients was 7.56 (IQR: 4.48–11.40) ng/ml. The rates of 25-(OH)D deficiency, insufficiency, and sufficiency were 67.1% (108/161), 29.2% (47/161), and 3.7% (6/161), respectively. Serum 25-(OH)D level was negatively correlated with alkaline phosphatase (*r* = ‐0.232, *P* = 0.003). After adjusting for age, a decrease in serum 25-(OH)D level was correlated with the increase of the *Batts-Ludwig* stage score (odds ratio (OR): 0.94, 95% confidence interval (CI): 0.88–0.99; *P* = 0.028). Serum 25-(OH)D level was also correlated with the N-terminal propeptide of type III procollagen (PIIINP) (*r* = ‐0.246, *P* = 0.002). Additionally, PIIINP (*P* = 0.038) and ALP (*P* = 0.031) were independently associated with serum 25-(OH)D level.

**Conclusions:**

VD deficiency was common and inversely correlated with liver fibrosis in BA patients. Furthermore, VD was not correlated with liver function except alkaline phosphatase.

## 1. Introduction

Biliary atresia (BA) is a rare disease of infancy, and patients present with jaundice and pale stools in the first few weeks of life [1]. The majority of patients develop progressive liver fibrosis and liver failure, even after Kasai hepatoportoenterostomy (KPE), leading to end-stage liver disease and require to undergo liver transplantation during childhood. Furthermore, severe fibrosis at the time of KPE predicted significantly worse survival of the native liver in infants with BA [2]. Effective strategies to prevent and treat liver damage and fibrosis may improve the prognosis of BA patients. Although the recent advances have enhanced our understanding about the pathogenesis and clinical management of BA, treatment remains a major challenge.

Vitamin D (VD) is involved in the regulation of bone and calcium homeostasis; however, VD has been recently demonstrated to possess pleiotropic biological actions in different cells and types of tissues [3]. VD deficiency is frequent in chronic liver diseases (CLDs) [4]. A number of scholars have focused on the potential associations between VD deficiency and liver disease, especially the relationships with liver function and liver fibrosis. VD is inversely associated with aspartate aminotransferase (AST) and aspartate aminotransferase (ALT) in patients with nonalcoholic fatty liver disease (NAFLD) [5]. High-dose VD supplementation may improve markers of liver function in adolescents with abnormalities in liver function tests [6]. Additionally, low circulating levels of VD associate with the development of hepatic fibrosis in patients with various CLDs (e.g., NAFLD and chronic hepatitis C virus (HCV)) [7–9]. These data suggest that VD deficiency plays a vital role in the progression of liver disease.

VD deficiency is frequent in BA patients [10, 11]. Although bile flow could be established after surgery, observation of VD deficiency in the majority of BA patients was confirmed [10], suggesting that VD deficiency contributes to the progression of liver function and liver fibrosis. The aim of the study was to evaluate serum 25-(OH)D level in a cohort of BA patients and investigate the association between serum 25-(OH)D level and liver function and liver fibrosis. The findings may provide a reliable reference for pathophysiology of BA and assist clinical VD supplementation and monitoring of BA patients.

## 2. Methods

### 2.1. Patients and Inclusion Criteria

A retrospective study was conducted on patients who were admitted to Children's Hospital of Fudan University (Shanghai, China) due to jaundice between January 2017 and February 2019. Pediatric patients with BA confirmed by intraoperative cholangiography were included in the present study. The exclusion criteria were as follows: (1) cholestasis without BA was confirmed by intraoperative cholangiography, (2) the age at the time of surgery was more than 180 days, and (3) there were missing data of serum 25-(OH)D level, Batts-Ludwig stage scores, or serum biomarker levels [N-terminal propeptide of type III procollagen (PIIINP), collagen type IV (CIV), laminin (LN), and hyaluronic acid (HA)].

### 2.2. Research Methods

In this study, collected data included demographic information (sex, weight, and age at the time of surgery), preoperative serum 25-(OH)D level, serum biomarkers of liver fibrosis (PIIINP, CIV, LV, HA), liver function, and Batts-Ludwig stage score. All serum 25-(OH)D concentrations were measured by electrochemiluminescence immunoassay (ECLIA) using a cobas e 602 module, and a vitamin D total kit was purchased from Roche Diagnostics GmbH (Mannheim, Germany). The coefficients of variation (CV) of intra-assay and intermediate precision were ≤6.8% and ≤13.1%, respectively.

To assess the status of VD deficiency, deficiency, insufficiency, and sufficiency were defined as serum 25-(OH)D levels of <10, 10-20, and >20 ng/ml, respectively [11]. Seasons were defined as spring (March, April, and May), summer (June, July, and August), autumn (September, October, and November), and winter (December, January, and February).

The Institutional Review Board and Ethics Committee of Children's Hospital of Fudan University approved the study protocol and waived the need for parents' informed consent due to the retrospective nature of the study design.

### 2.3. Statistical Analysis

The Shapiro-Wilk test was performed for all continuous variables. Discrete variables were presented using frequencies and percentages. Continuous variables were expressed as the mean ± standard deviation (SD) or the median (interquartile range (IQR)), as appropriate. Student's *t*-test was used to compare continuous variables between the two groups. Correlations between variables were analyzed by using Spearman's correlation analysis, partial correlation analysis (correcting for age), or ordinal logistic regression analysis, as appropriate. Univariate and multivariate linear regression analyses were utilized to assess the association between serum 25-(OH)D level and variables. Variables with *P* < 0.1 in univariate analysis were tested in multivariate regression analysis. Multivariate models were obtained by backward selection, using a *P* value > 0.10 for removal from the model. A *P* value < 0.05 was considered statistically significant. All statistical analyses were performed by using SPSS 20.0 software (IBM, Armonk, NY, USA).

## 3. Results

### 3.1. Patients' Demographic and Clinical Characteristics

According to the above-defined inclusion criteria, 161 BA infants confirmed by intraoperative cholangiography who were admitted to Children's Hospital of Fudan University between January 2017 and February 2019 were recruited in the present study. The baseline demographic and clinical characteristics of the included patients are shown in Table 1. There were statistically significant differences in PIIINP (*P* = 0.041) and ALP (*P* = 0.007) among the three groups.

### 3.2. Preoperative VD Status and Univariate and Multivariate Linear Regression Analyses of Factors Associated with Serum 25-(OH)D Level

The median (interquartile range (IQR)) serum 25-(OH)D level in all the patients was 7.56 (IQR 4.48–11.40) ng/ml. The prevalence of 25-(OH)D deficiency, insufficiency, and sufficiency was 67.1% (108/161), 29.2% (47/161), and 3.7% (6/161), respectively. PIIINP, ALP, and calcium were tested by multivariate linear regression analysis. PIIINP (*P* = 0.038) and ALP (*P* = 0.031) were independently associated with serum 25-(OH)D level (Table 2).

### 3.3. Relationship between Serum 25-(OH)D Level and Liver Function in BA Patients

Table 3 illustrates the correlation between serum 25-(OH)D level and liver function. Serum 25-(OH)D level was correlated with ALT (*r* = 0.158, *P* = 0.045), AST (*r* = 0.182, *P* = 0.021), and ALP (*r* = ‐0.232, *P* = 0.003) (Table 3). For those variables correlated with age (GGT, ALT, AST, TCL, TBA, and phosphate), partial correlation analyses controlling age were performed. Those analyses revealed that ALT and AST were not correlated with serum 25-(OH)D level (Table 3).

### 3.4. Relationship between Serum 25-(OH)D Level and Liver Fibrosis in BA Patients

#### 3.4.1. Relationship between Serum 25-(OH)D Level and Stage Scores in BA Patients

Stage scores were correlated with age (*r* = 0.536, *P* < 0.001), while ordinal logistic regression analysis disclosed that serum 25-(OH)D level was not correlated with the stage score (*P* = 0.143). After adjusting for age, a decrease in serum 25-(OH)D level was resulted in the increase of the stage score (odds ratio (OR): 0.94, 95% confidence interval (CI): 0.88-0.99; *P* = 0.028).

#### 3.4.2. Relationship between Serum 25-(OH)D Level and Preoperative Biomarkers of Liver Fibrosis in BA Patients

It was uncovered that PIIINP (*r* = 0.345, *P* < 0.001), CIV (*r* = 0.312, *P* < 0.001), and HA (*r* = 0.452, *P* < 0.001) were increased with the elevation of age, suggesting the development of significant fibrosis before surgery. No correlation between LN and age was observed (*r* = 0.007, *P* = 0.929). Serum 25-(OH)D level was correlated with only PIIINP (*r* = ‐0.246, *P* = 0.002), rather than CIV (*r* = ‐0.033, *P* = 0.680), LN (*r* = ‐0.079, *P* = 0.319), or HA (*r* = 0.10, *P* = 0.890). After adjusting for age, partial correlation analyses showed that 25-(OH)D level was correlated with only PIIINP (*r* = ‐0.212, *P* = 0.007), but not CIV (*r* = ‐0.120, *P* = 0.131), LN (*r* = ‐0.092, *P* = 0.250), or HA (*r* = ‐0.145, *P* = 0.068).

#### 3.4.3. Relationship between Serum 25-(OH)D Level and Grade Scores in BA Patients

Grade scores were correlated with age (*r* = 0.236, *P* = 0.003), while ordinal logistic regression analysis revealed that serum 25-(OH)D level was not correlated with grade scores (*P* = 0.157). After adjusting for age, there was no correlation between 25-(OH)D level and grade scores (*P* = 0.181).

## 4. Discussion

VD deficiency is frequent in children and adults and leads to serious health problems worldwide. In recent years, VD has notably attracted scholars' attention because of its influences on cell growth and differentiation, immune function, and cardiovascular function, as well as calcium and phosphate homeostasis [12]. Researchers have also investigated the effect of VD deficiency on hepatic pathophysiology. A number of scholars showed that VD deficiency was common in different liver diseases [13]. Furthermore, low VD level was associated with the severity of liver fibrosis [3]. In this study, we attempted to assess the relationship between VD deficiency and liver function and liver fibrosis in BA patients. We found that serum 25-(OH)D level was inversely associated with the severity of liver fibrosis and serum PIIINP level in BA patients, suggesting that VD had a protective influence on liver fibrosis. However, with the exception of ALP, no correlation was noted between serum 25-(OH)D level and liver function.

VD deficiency is generally defined as a 25-(OH)D level of less than 20 ng/ml, which is widely believed to be suboptimal for an individual's health [14]. However, the levels of VD insufficiency and deficiency were not clearly defined in children. The British Paediatric and Adolescent Bone Group defined deficiency and insufficiency of VD as 25-(OH)D level of <10 and 10-20 ng/ml, respectively [15]. This definition was used in the present study as it was also in agreement with other reports related to VD deficiency in BA patients [11]. Serum 25-(OH)D level was associated with age and latitude. Serum 25-(OH)D level was 29 ± 11 ng/ml in healthy infants who aged 0-3 months in southeast China [16]. In Shanghai (31° N latitude), the median serum 25-(OH)D concentration was reported 22.4 ng/ml in newborns and 20.9 ng/ml in adults [17, 18]. However, median serum 25-(OH)D concentration was 7.56 ng/ml and the prevalence of 25-(OH)D deficiency was 67.1% in the current study, which revealed that VD deficiency was frequent and severe in BA patients. This phenomenon was also reported in previous researches [10, 11]. Such findings indicate that preoperative VD supplementation is necessary for young BA infants with early clinical diagnosis.

There are few data detailing the correlation between VD and liver function in BA patients. In the present study, no association was observed between serum 25-(OH)D level and the majority of biomarkers of liver function, with the exception of ALP. Ng et al. [11] also found negative correlations between serum 25-(OH)D concentration and ALP in BA infants both preoperatively and postoperatively. Elevated ALP levels indicate that there could be a disease that affects blood calcium level (hyperparathyroidism), VD deficiency, or damaged liver cells [19]. ALP is closely associated with bone metabolism in postoperative BA patients [20]. The effects of VD on liver function are controversial. In the current research, we did not observe an association between serum 25-(OH)D concentration and bilirubin level in BA patients. Bilirubin level indicates the severity of cholestasis and resultant fat malabsorption. Ng et al. [11] found correlations between 25-(OH)D concentration and bilirubin level, pre-KPE and 6 and 12 months post-KPE in BA patients. However, such correlation was not found at 1 or 4 months post-KPE [11]. The serum 25-(OH)D level was significantly higher in the jaundice-resolved group compared with the jaundice-unresolved group in BA patients [10]. Such conflicting results suggested that serum 25-(OH)D level was influenced by multiple factors aside from bilirubin, such as dietary intake or polymorphisms in VD hydroxylase enzymes. After adjusting for age, AST and ALT were not correlated with serum 25-(OH)D level. Recently, a meta-analysis showed that VD supplementation had no influence on liver function, e.g., AST and ALT, in patients with NAFLD [21].

Progressive liver fibrosis is a prominent feature of BA and is also the most important predictor of outcome following KPE. VD deficiency was found to be frequent in BA patients, even after KPE surgery and oral VD supplementation [10]. Thus, VD deficiency may be involved in the progression of liver fibrosis in BA patients. In recent years, a number of studies revealed that VD deficiency is associated with the severity of liver fibrosis in patients with liver diseases, involving CLDs [7] and chronic hepatitis C [22]. There are limited data presenting an association between VD deficiency and liver fibrosis in BA patients. In the present research, we found that VD deficiency was associated with the severity of liver fibrosis in BA patients preoperatively, suggesting that VD deficiency was involved in disease progression. Homchan et al. [20] found that serum 25-(OH)D level in postoperative BA patients with jaundice was lower than those in patients without jaundice. However, there were no data regarding liver fibrosis in that study. Peng et al. [23] demonstrated that there was an inverse correlation between serum 25-(OH)D level and liver fibrosis in BA patients post-KPE. There were some differences between their study and ours. Firstly, they used acoustic radiation force impulse (ARFI) to assess liver fibrosis. However, liver histology is the most accurate method to evaluate the severity of liver fibrosis. We used both liver histology (Batts-Ludwig stage scores) and four liver fibrosis biomarkers to assess the severity of liver fibrosis in our study. Secondly, patients' age widely ranged from 1 to 23 years in their study. However, age has an effect on serum 25-(OH)D. Although no correlation between age and 25-(OH)D was found, it might be explained by the small sample size. In our study, the age of patients was similar. Thirdly, we assessed an association between VD and liver fibrosis at KPE, rather than post-KPE, and emphasized the importance of VD deficiency on liver fibrosis in the early stage of BA. Finally, Peng et al. excluded the patients who had received liver transplantation, which might cause some bias.

In the present research, we used the Batts-Ludwig grading system to assess liver inflammation and fibrosis [24]. Liver inflammation is a hallmark of BA and is related to the progression of liver fibrosis. No correlation was observed between serum 25-(OH)D level and grade score, which may be explained by the skewed distribution of grade scores. Additionally, PIIINP, CIV, LN, and HA are the four major biomarkers of liver fibrosis that reflect the deposition or removal of ECM. In the present study, all four biomarkers were significantly elevated in BA patients. We also found that PIIINP, CIV, and HA were correlated with the severity of liver fibrosis in preoperative BA patients (data were not shown). However, only PIIINP was found to be inversely associated with 25-(OH)D level in our study. After adjusting for age, a trend toward an inverse correlation between serum 25-(OH)D level and CIV, LN, and HA was observed, although no statistical significance was found. This may be explained by several reasons. Firstly, the sample size might not be big enough to reach a statistical difference. Secondly, there might be other factors which influence the serum levels of 25-(OH)D on serum fibrosis markers except age. Thirdly, the mechanism that VD alleviates liver fibrosis is not fully understood. These four liver fibrosis markers might be regulated through different pathways. It is known that expression of collagens I and III could be suppressed by VD via inhibiting the activation of the HSC-mediated transforming growth factor beta (TGF-*β*) signaling pathway [3].

Multiple researches explored the mechanisms of VD on liver fibrosis. VD can reduce the expression of collagen and key profibrotic factors in hepatic stellate cells (HSCs), LX-2 cells, and mesenchymal multipotent cells (MMCs) [25–27]. The administration of VD can significantly reduce deposition of extracellular matrix (ECM) and attenuate the fibrotic score in different animal models of hepatic injury [26, 28, 29]. VDR knockout mice develop spontaneous liver fibrosis, demonstrating that the VD/VDR signaling pathway regulates hepatic fibrogenesis [26]. Activated HSCs play a critical role in liver fibrogenesis [30]. VD has antiproliferative and antifibrotic properties in HSCs by inhibiting the TGF-*β*/SMAD signaling pathway [28], which is one of the most potent profibrogenic pathways in the liver. The VD-VDR complex can function as a ligand-dependent transcriptional repressor within HSCs, antagonizing TGF-*β*-mediated recruitment of SMAD3 to the regulatory loci of numerous profibrotic genes.

There are some limitations in our study. Firstly, there was a lack of data on potential confounders that may influence the level of VD, including exposure to sunshine, dietary intake, prevalence of osteoporosis, polymorphism of VD hydroxylase enzymes, parathyroid hormones [31], and VD-binding protein [32]. VD levels are regulated by cascade genes. Polymorphisms of related genes have been found to be associated with serum VD levels and treatment response, which should be taken into account in the future study. Secondly, this was a retrospective study with cross-sectional data, which cannot provide causation. Although with a limitation, we believe that this was a very interesting and meaningful finding which suggested that VD has an antifibrosis effect on BA patients before surgery. A well-designed prospective study will enhance the reliability of the results. Thirdly, there was a lack of the long-term follow-up data, such as postoperative serum 25-(OH)D level and mortality rate. Thus, the relationship of VD deficiency and morbidity and mortality rate could not be determined, which would be of paramount importance. Lastly, all the patients did not receive routine VD supplements before surgery. One vitamin AD gel capsule (each containing 2000 IU of vitamin A and 2700 IU of vitamin D) was administered daily beginning on postoperative day 4 (resumption of oral diet) after surgery in our institution. However, related outcomes such as serum levels of VDR and fibrogenic factors were not available postoperatively. Thus, the effect of VD supplementation on outcomes could not be determined. Since serum 25-(OH)D levels were very low at presentation, VD supplements should be advised before surgery. Also, serum levels of VDR and fibrogenic factors should be examined postoperatively in the future.

In conclusion, our findings demonstrated low serum 25-(OH)D level in BA patients and an inverse correlation of VD status and the severity of liver fibrosis. Furthermore, with the exception of alkaline phosphatase, serum 25-(OH)D concentration was not correlated with liver function. There is progressive liver fibrosis before KPE; VD demonstrates its protective effect on liver fibrosis to a certain extent. Recently, VD supplementation was found to be an attractive therapeutic strategy for treating liver fibrosis, as well as improving liver function [6, 33]. Preoperative VD supplementation might be helpful for alleviating liver fibrosis in young infants with an early clinical diagnosis of BA. Further prospective studies are needed to determine the relationship of VD deficiency and morbidity and mortality rate, as well as the effect of VD supplementation on outcomes in BA patients.

## Figures and Tables

**Table 1:** Demographic and clinical characteristics of the BA patients. Data were presented as frequencies (percentages), mean ± SD, or median (interquartile range [IQR]), as appropriate.

Variable	25-(OH)D sufficiency (*n* = 6)	25-(OH)D insufficiency (*n* = 47)	25-(OH)D deficiency (*n* = 108)	*P* value
25-(OH)D	24.6 (22.8, 29.6)	12.4 (11.2, 14.5)	5.8 (3.6, 7.6)	<0.001
Age (days)	75 (57, 90)	63 (51, 80)	63 (53, 76)	0.508
Sex (male/female)	4/2	21/26	55/53	0.490
Weight (kg)	5.0 ± 0.8	4.7 ± 0.9	4.7 ± 0.9	0.619
GGT (IU/l)	658.5 (160.8, 1023.5)	514.0 (349.0, 789.9)	474.0 (229.0, 847.0)	0.793
Albumin (g/l)	38.4 ± 4.1	38.8 ± 3.6	38.8 ± 3.1	0.911
ALT (IU/l)	128.5 (80.0, 160.3)	109.0 (67.0, 191.0)	95.0 (73.8, 147.0)	0.442
AST (IU/l)	229.0 (104.3, 266.8)	172.0 (123.0, 241.0)	162.5 (113.3, 217.2)	0.595
TB (*μ*mol/l)	157.0 (123.8, 183.5)	153.0 (122.0, 186.0)	155.5 (125.5, 181.3)	0.998
DB (*μ*mol/l)	92.0 (78.5, 109.0)	97.2 (82.2, 127.0)	96.7 (82.0, 119.8)	0.645
ALP (IU/l)	479.5 (404.8, 571.0)	528.0 (443.0, 620.0)	606.0 (475.0, 756.5)	0.007
TCL (mmol/l)	5.0 (4.62, 6.25)	4.7 (4.0, 6.0)	5.0 (4.0, 5.4)	0.581
TBA (*μ*mol/l)	127.2 (96.1, 256.0)	122.8 (95.0, 161.0)	123.6 (100.8, 153.4)	0.910
Phosphate (mmol/l)	1.86 ± 0.26	1.84 ± 0.30	1.84 ± 0.28	0.980
Calcium (mmol/l)	2.33 ± 0.52	2.40 ± 0.48	2.30 ± 0.44	0.408
Batts-Ludwig stage score				0.459
0, *N* (%)	0 (0%)	0 (0%)	0 (0%)	
1, *N* (%)	0 (0%)	0 (0%)	0 (0%)	
2, *N* (%)	3 (50%)	21 (44.7%)	33 (30.6%)	
3, *N* (%)	2 (33%)	18 (38.3%)	55 (50.9%)	
4, *N* (%)	1 (17%)	8 (17.0%)	20 (18.5%)	
Batts-Ludwig grade score				0.539
0, *N* (%)	0 (0%)	0 (0%)	0 (0%)	
1, *N* (%)	0 (0%)	0 (0%)	3 (2.8%)	
2, *N* (%)	0 (0%)	9 (19.1%)	22 (20.4%)	
3, *N* (%)	6 (100%)	38 (80.9%)	83 (76.9%)	
4, *N* (%)	0 (0%)	0 (0%)	0 (0%)	
PIIINP (ng/ml)	547.9 (306.2, 820.3)	371.2 (266.3, 508.2)	467.9 (318.0, 678.1)	0.041
CIV (ng/ml)	578.0 (425.9, 1027.7)	517.9 (376.2, 800.4)	565.3 (398.9, 916.9)	0.450
LN (ng/ml)	354.1 (154.5, 389.7)	352.1 (241.2, 449.6)	330.1 (245.4, 473.8)	0.777
HA (ng/ml)	212.4 (129.4, 459.5)	208.3 (160.5, 283.2)	218.1 (163.5, 328.1)	0.769
Season				0.050
Spring	1 (16.7%)	17 (36.2%)	48 (44.4%)	
Summer	1 (16.7%)	17 (36.2%)	27 (25.0%)	
Autumn	2 (33.3%)	1 (5.9%)	6 (5.6%)	
Winter	2 (33.3%)	12 (25.5%)	27 (25.0%)	

GGT: gamma-glutamyl transferase; ALT: alanine transaminase; AST: aspartate transaminase; TB: total bilirubin; DB: direct bilirubin; ALP: alkaline phosphatase; TCL: total cholesterol; TBA: total bile acids; PIIINP: N-terminal propeptide of type III procollagen; CIV: collagen type IV; LN: laminin; HA: hyaluronic acid.

**Table 2:** Linear regression analysis of serum 25-(OH)D level in BA patients.

Variable	Univariate			Multivariate		
	Beta	BE	*P* value	Beta	BE	*P* value
Age	0.018	0.022	0.416			
Weight	0.383	0.468	0.431			
GGT	0.000	0.001	0.931			
Albumin	0.101	0.132	0.449			
ALT	0.006	0.005	0.224			
AST	0.006	0.004	0.143			
TB	0.002	0.009	0.863			
DB	0.000	0.014	0.991			
ALP	-0.004	0.002	0.023	-0.004	0.002	0.031
TCL	-0.029	0.222	0.897			
TBA	0.006	0.007	0.390			
Phosphate	0.499	1.534	0.745			
Calcium	1.827	0.944	0.055			
Batts-Ludwig grade score	1.393	0.931	0.136			
Batts-Ludwig stage score	-0.842	0.607	0.168			
PIIINP	-0.002	0.001	0.028	-0.002	0.001	0.038
CIV	-0.001	0.001	0.238			
LN	-0.003	0.003	0.251			
HA	-0.002	0.001	0.209			
Season	0.331	0.358	0.357			

GGT: gamma-glutamyl transferase; ALT: alanine transaminase; AST: aspartate transaminase; TB: total bilirubin; DB: direct bilirubin; ALP: alkaline phosphatase; TCL: total cholesterol; TBA: total bile acids; PIIINP: N-terminal propeptide of type III procollagen; CIV: collagen type IV; LN: laminin; HA: hyaluronic acid.

**Table 3:** Correlation of serum 25(OH)D level with liver function.

Variable	Correlation coefficient (*r*)	*P* value	Partial correlation coefficient (*r*_p_)	*P* value
GGT	0.052	0.514	-0.008	0.918
Albumin	0.060	0.449		
ALT	0.158	0.045	0.105	0.186
AST	0.182	0.021	0.081	0.311
TB	0.069	0.386		
DB	0.065	0.415		
ALP	-0.232	0.003		
TCL	-0.009	0.910	-0.031	0.697
TBA	-0.018	0.822	0.042	0.599
Phosphate	0.026	0.745	0.051	0.523
Calcium	0.152	0.055		

GGT: gamma-glutamyl transferase; ALT: alanine transaminase; AST: aspartate transaminase; TB: total bilirubin; DB: direct bilirubin; ALP: alkaline phosphatase; TCL: total cholesterol; TBA: total bile acids.

## Data Availability

The data used to support the findings of this study are available from the corresponding author upon request.

## References

[1] Kelly D. A., Davenport M. (2007). Current management of biliary atresia. *Archives of Disease in Childhood*.

[2] Weerasooriya V. S., White F. V., Shepherd R. W. (2004). Hepatic fibrosis and survival in biliary atresia. *The Journal of Pediatrics*.

[3] Udomsinprasert W., Jittikoon J. (2019). Vitamin D and liver fibrosis: molecular mechanisms and clinical studies. *Biomedicine & Pharmacotherapy*.

[4] Arteh J., Narra S., Nair S. (2010). Prevalence of vitamin D deficiency in chronic liver disease. *Digestive Diseases and Sciences*.

[5] Izadi A., Aliasghari F., Gargari B. P., Ebrahimi S. (2019). Strong association between serum vitamin D and vaspin levels, AIP, VAI and liver enzymes in NAFLD patients. *International Journal for Vitamin and Nutrition Research*.

[6] Tavakoli H., Rostami H., Avan A. (2019). High dose vitamin D supplementation is associated with an improvement in serum markers of liver function. *BioFactors*.

[7] Ko B. J., Kim Y. S., Kim S. G. (2016). Relationship between 25-hydroxyvitamin D levels and liver fibrosis as assessed by transient elastography in patients with chronic liver disease. *Gut and Liver*.

[8] Kim H.-S., Rotundo L., Kothari N., Kim S.-H., Pyrsopoulos N. (2017). Vitamin D is associated with severity and mortality of non-alcoholic fatty liver disease: a US population-based study. *Journal of Clinical and Translational Hepatology*.

[9] Avihingsanon A., Jitmitraparp S., Tangkijvanich P. (2014). Advanced liver fibrosis by transient elastography, fibrosis 4, and alanine aminotransferase/platelet ratio index among Asian hepatitis C with and without human immunodeficiency virus infection: role of vitamin D levels. *Journal of Gastroenterology and Hepatology*.

[10] Dong R., Sun S., Liu X. Z., Shen Z., Chen G., Zheng S. (2017). Fat-soluble vitamin deficiency in pediatric patients with biliary atresia. *Gastroenterology Research and Practice*.

[11] Ng J., Paul A., Wright N., Hadzic N., Davenport M. (2016). Vitamin D levels in infants with biliary atresia: pre- and post-Kasai portoenterostomy. *Journal of Pediatric Gastroenterology and Nutrition*.

[12] Christakos S., Dhawan P., Verstuyf A., Verlinden L., Carmeliet G. (2016). Vitamin D: metabolism, molecular mechanism of action, and pleiotropic effects. *Physiological Reviews*.

[13] Elangovan H., Chahal S., Gunton J. E. (2017). Vitamin D in liver disease: current evidence and potential directions. *Biochimica et Biophysica Acta (BBA) - Molecular Basis of Disease*.

[14] Holick M. F. (2007). Vitamin D deficiency. *New England Journal of Medicine*.

[15] Arundel P., Ahmed S. F., Allgrove J. (2012). British paediatric and adolescent bone group’s position statement on vitamin D deficiency. *BMJ*.

[16] Wang S., Shen G., Jiang S. (2017). Nutrient status of vitamin D among Chinese children. *Nutrients*.

[17] Yu X., Wang W., Wei Z. (2014). Vitamin D status and related factors in newborns in Shanghai, China. *Nutrients*.

[18] Lu H.-K., Zhang Z., Ke Y.-H. (2012). High prevalence of vitamin D insufficiency in China: relationship with the levels of parathyroid hormone and markers of bone turnover. *PLoS One*.

[19] Sharma U., Pal D., Prasad R. (2014). Alkaline phosphatase: an overview. *Indian Journal of Clinical Biochemistry*.

[20] Homchan K., Chaiwatanarat T., Udomsinprasert W., Chongsrisawat V., Poovorawan Y., Honsawek S. (2017). Low bone mineral density and the severity of cholestasis in biliary atresia. *World Journal of Hepatology*.

[21] Tabrizi R., Moosazadeh M., Lankarani K. B. (2017). The effects of vitamin D supplementation on metabolic profiles and liver function in patients with non-alcoholic fatty liver disease: a systematic review and meta-analysis of randomized controlled trials. *Diabetes & Metabolic Syndrome: Clinical Research & Reviews*.

[22] Petta S., Cammà C., Scazzone C. (2010). Low vitamin D serum level is related to severe fibrosis and low responsiveness to interferon-based therapy in genotype 1 chronic hepatitis C. *Hepatology*.

[23] Peng C.-H., Lee H.-C., Jiang C.-B. (2019). Serum vitamin D level is inversely associated with liver fibrosis in post Kasai’s portoenterostomy biliary atresia patients living with native liver. *PLoS One*.

[24] Batts K. P., Ludwig J., Chronic h. (1995). An update on terminology and reporting. *The American Journal of Surgical Pathology*.

[25] Artaza J. N., Norris K. C. (2009). Vitamin D reduces the expression of collagen and key profibrotic factors by inducing an antifibrotic phenotype in mesenchymal multipotent cells. *The Journal of Endocrinology*.

[26] Ding N., Yu R. T., Subramaniam N. (2013). A vitamin D receptor/SMAD genomic circuit gates hepatic fibrotic response. *Cell*.

[27] Beilfuss A., Sowa J.-P., Sydor S. (2015). Vitamin D counteracts fibrogenic TGF-*β* signalling in human hepatic stellate cells both receptor-dependently and independently. *Gut*.

[28] Abramovitch S., Dahan-Bachar L., Sharvit E. (2011). Vitamin D inhibits proliferation and profibrotic marker expression in hepatic stellate cells and decreases thioacetamide-induced liver fibrosis in rats. *Gut*.

[29] Hochrath K., Stokes C. S., Geisel J. (2014). Vitamin D modulates biliary fibrosis in ABCB4-deficient mice. *Hepatology International*.

[30] Tsuchida T., Friedman S. L. (2017). Mechanisms of hepatic stellate cell activation. *Nature Reviews Gastroenterology & Hepatology*.

[31] Khundmiri S. J., Murray R. D., Lederer E. (2016). PTH and Vitamin D. *Comprehensive Physiology*.

[32] Yousefzadeh P., Shapses S. A., Wang X. (2014). Vitamin D binding protein impact on 25-hydroxyvitamin D levels under different physiologic and pathologic conditions. *International Journal of Endocrinology*.

[33] Komolmit P., Kimtrakool S., Suksawatamnuay S. (2017). Vitamin D supplementation improves serum markers associated with hepatic fibrogenesis in chronic hepatitis C patients: a randomized, double-blind, placebo-controlled study. *Scientific Reports*.

